# Supplementary material for: Content-rich biological network constructed by mining PubMed abstracts
Source: BMC Bioinformatics. 2004 Oct 8;5:147. doi: 10.1186/1471-2105-5-147 (PMC528731; doi:10.1186/1471-2105-5-147)
Supplement: Additional File 2 — The original results of the above study (non-essential files are deleted to keep the file size under the limit set by BMC bioinformatics). [file 1471-2105-5-147-S2.bz2 › chilibotAdditionalFile2/dip05/336ID10768840E3972/html/TNFSF8_TNFRSF8.html]

 


 **TNFSF8** and **TNFRSF8** 
  
Found 158 abstracts in PubMed, retrieved 05.  
 

 What does Google say? 
 PDF only 
| .edu only 

---

**Interactive relationship** (e.g. stimulation, inhibition, etc)

**Inhibitory relationship**- Proliferation of CD30  [ **TNFRSF8** ]  T Helper 2 Lymphoma Cells Can Be Inhibited by CD30  [ **TNFRSF8** ]  Receptor Cross Linking with Recombinant CD30  [ **TNFRSF8** ]  Ligand  [ **TNFSF8** ] .  Ref: 12855655 Clin Cancer Res, 2003
**Stimulatory relationship**- PURPOSE Cutaneous anaplastic large cell lymphomas ALCLs are characterized by the expression of CD30  [ **TNFRSF8** ] , spontaneous regression of skin lesions, and increased concentration of CD30  [ **TNFRSF8** ]  ligand  [ **TNFSF8** ]  CD30L  [ **TNFSF8** ] .  Ref: 12855655 Clin Cancer Res, 2003
**Neutral relationship**- Comparison of effects of ligation with CD30L  [ **TNFSF8** ]  and anti CD30  [ **TNFRSF8** ]  agonistic antibody HeFi 1 revealed higher efficacy for CD30L  [ **TNFSF8** ]  in these ALCL lines.  Ref: 12855655 Clin Cancer Res, 2003
- The activation antigen CD30  [ **TNFRSF8** ]  Ki 1  [ **TNFRSF8** ]  which is expressed by the lymphocytes T and B is related to the nerve growth factor NFGR like the receptor of the tumor necrosis factor  [ **TNFSF8** ]  TNF and is expressed in the group of large cell anaplastic lymphoma ACLC and Hodgkin s lymphoma and in non Hodgkin s lymphoma and within the T cells of infectious Mononucleosis.  Ref: 12730648 Infez Med< MedlineTA>Infez Med, 2003

**Non-interactive relationship** (e.g. studied together, co-existance, homology, etc.)

- Co expression of IL 6 and IL 6R in the epithelial follicular cells was observed solely in CD30L  [ **TNFSF8** ]  CD30  [ **TNFRSF8** ]  positive nodules 5 15 33% oncocytic adenomas.  Ref: 12553555 J Endocrinol Invest, 2002
- All 3 non metastasized MTC were IL 6 IL 6R negative, and 1 3 was CD30L  [ **TNFSF8** ]  positive CD30  [ **TNFRSF8** ]  negative.  Ref: 12553555 J Endocrinol Invest, 2002
- We hypothesize that CD30  [ **TNFRSF8** ]  CD30L  [ **TNFSF8** ]  interactions explain the unusual clinical behavior of cutaneous ALCLs.  Ref: 12855655 Clin Cancer Res, 2003
- In the normal thyroid tissue, colloid nodules, and another subset of benign and malignant thyroid nodules, IL 6 expression is under control of signals other than CD30L  [ **TNFSF8** ]  CD30  [ **TNFRSF8** ] .  Ref: 12553555 J Endocrinol Invest, 2002
- Co expression of interleukin 6 IL 6 and interleukin 6 receptor IL 6R in thyroid nodules is associated with co expression of CD30  [ **TNFRSF8** ]  ligand  [ **TNFSF8** ]  CD30  [ **TNFRSF8** ]  receptor.  Ref: 12553555 J Endocrinol Invest, 2002
- 14 15 FTC and 5 6 ATC were CD30L  [ **TNFSF8** ]  CD30  [ **TNFRSF8** ]  negative.  Ref: 12553555 J Endocrinol Invest, 2002
- Unlike dendritic cells, the CD4 CD3 cells have little CD80 or CD86 expression but do express high levels of the TNF ligands, OX40 ligand and CD30  [ **TNFRSF8** ]  ligand  [ **TNFSF8** ] .  Ref: 12753741 Immunity, 2003
- Based on our recent data of CD30  [ **TNFRSF8** ]  ligand  [ **TNFSF8** ]  CD30L  [ **TNFSF8** ]  CD30  [ **TNFRSF8** ]  receptor CD30  [ **TNFRSF8** ]  in these nodules and on the knowledge that this signal stimulates IL 6 production in non thyroid neoplasms, we wanted to evaluate the immunocytochemical expression of these 2 ligand receptor systems in a large archival series of paraffin embedded specimens.  Ref: 12553555 J Endocrinol Invest, 2002
- IL 6 staining associated to absent expression of CD30L  [ **TNFSF8** ]  and CD30  [ **TNFRSF8** ]  was observed in 7 follicular adenomas all belonging to variants different from toxic and hyalinizing trabecular, 2 oncocytic adenomas, 5 of the 30 colloid nodules and 2 normal thyroids.  Ref: 12553555 J Endocrinol Invest, 2002
- Of the 6 tumors arising from the parafollicular C cells medullary thyroid cancer, MTC, all 3 that had metastasized were CD30L  [ **TNFSF8** ]  CD30  [ **TNFRSF8** ]  IL 6 positive and IL 6R negative.  Ref: 12553555 J Endocrinol Invest, 2002
- We conclude that only in a subset of both benign and malignant thyroid nodules the IL 6 IL 6R signal could be induced by the CD30L  [ **TNFSF8** ]  CD30  [ **TNFRSF8** ] .  Ref: 12553555 J Endocrinol Invest, 2002
- In another oncocytic adenoma, another 4 conventional PTC and another 7 non conventional PTC CD30L  [ **TNFSF8** ]  CD30  [ **TNFRSF8** ]  expression was associated to expression of IL 6 only.  Ref: 12553555 J Endocrinol Invest, 2002
- In a cell kinetic analysis we found that ALCL cell growth is effectively inhibited by CD30L  [ **TNFSF8** ]  but only in those cell lines expressing CD30  [ **TNFRSF8** ]  molecules in sufficient amounts on the cell surface.  Ref: 12855655 Clin Cancer Res, 2003
